# Exploring the Regulatory Mechanism of *Hedysarum Multijugum Maxim.*-*Chuanxiong Rhizoma* Compound on HIF-VEGF Pathway and Cerebral Ischemia-Reperfusion Injury’s Biological Network Based on Systematic Pharmacology

**DOI:** 10.3389/fphar.2021.601846

**Published:** 2021-06-25

**Authors:** Kailin Yang, Liuting Zeng, Anqi Ge, Yi Chen, Shanshan Wang, Xiaofei Zhu, Jinwen Ge

**Affiliations:** ^1^ Key Laboratory of Hunan Province for Integrated Traditional Chinese and Western Medicine on Prevention and Treatment of Cardio-Cerebral Diseases, Hunan University of Chinese Medicine, Changsha, China; ^2^ Galactophore Department, The First Hospital of Hunan University of Chinese Medicine, Changsha, China; ^3^ School of Graduate, Central South University, Changsha, China; ^4^ Shaoyang University, Shaoyang, China

**Keywords:** hedysarum multijugum maxim-chuanxiong rhizoma compound, cerebral ischemia-reperfusion injury, Ischemic stroke, cerebral ischemia, HIF-VEGF pathway, systematic pharmacology, chinese medicine

## Abstract

**Background:** Clinical research found that *Hedysarum Multijugum Maxim.*-*Chuanxiong Rhizoma* Compound (HCC) has definite curative effect on cerebral ischemic diseases, such as ischemic stroke and cerebral ischemia-reperfusion injury (CIR). However, its mechanism for treating cerebral ischemia is still not fully explained.

**Methods:** The traditional Chinese medicine related database were utilized to obtain the components of HCC. The Pharmmapper were used to predict HCC’s potential targets. The CIR genes were obtained from Genecards and OMIM and the protein-protein interaction (PPI) data of HCC’s targets and IS genes were obtained from String database. After that, the DAVID platform was applied for Gene Ontology (GO) enrichment analysis and pathway enrichment analysis. Finally, a series of animal experiments were carried out to further explore the mechanism of HCC intervention in CIR.

**Results:** The prediction results of systematic pharmacology showed that HCC can regulate CIR-related targets (such as AKT1, MAPK1, CASP3, EGFR), biological processes (such as angiogenesis, neuronal axonal injury, blood coagulation, calcium homeostasis) and signaling pathways (such as HIF-1, VEGF, Ras, FoxO signaling). The experiments showed that HCC can improve the neurological deficit score, decrease the volume of cerebral infarction and up-regulate the expression of HIF-1α/VEGF and VEGFR protein and mRNA (*p* < 0.05).

**Conclusion:** HCC may play a therapeutic role by regulating CIR-related targets, biological processes and signaling pathways found on this study.

## Introduction

Cerebrovascular disease is a common disease in the clinic, which seriously endangers human health and life. As the population of aging increases, the morbidity, mortality and disability rate of cerebrovascular disease were increasing year by year (Thomas, 1996; Sacco and Rundek, 2012; Liu et al., 2015). Among them, ischemic cerebrovascular disease accounts for a large proportion. The key to cerebral ischemia treatment is to quickly restore cerebral blood perfusion and maintain smooth blood flow (Frizzell, 2005; Behravan et al., 2014). However, the brain injury may be further aggravated after the restoration of blood flow perfusion, that is cerebral ischemia-reperfusion injury (CIR). Ischemia-reperfusion injury (IRI) refers to the pathological phenomenon that the degree of tissue damage is increased after the blood supply to the ischemic tissue is restored for a certain period of time (Wu et al., 2018). The harm of CIR is huge. It involves many complicated links and factors, which has been the focus of scientists’ research for many years. With the deepening of the research, while looking for neuroprotective drugs, a variety of comprehensive intervention strategies for CIR such as mild hypothermia, atmospheric hyperbaric therapy and ischemic preconditioning and ischemic postconditioning were also proposed (Lapi and Colantuoni, 2015; Patel and McMullen, 2017; Leech et al., 2019); the drugs include: N-methyl-D-aspartic acid (NMDA) receptor antagonist, Ca_2_ + channel blocker, ICAM-1 antibody, CDP-choline, and so on (Lapi and Colantuoni, 2015; Patel and McMullen, 2017). At present, natural plant ingredients have been found to improve microcirculation barriers after CIR, and Chinese medicine formulations have gradually become a new direction for new drug development (Wang et al., 2019).

*Hedysarum Multijugum Maxim.*-*Chuanxiong Rhizoma* Compound (HCC) was first used by the First Affiliated Hospital of Hunan University of Chinese Medicine. Clinical research showed that HCC has definite curative effect on cerebral ischemic diseases (such as ischemic stroke and CIR), and its clinical effective rate is over 90% (Ge, 2014). This Chinese medicine formula is composed of *Hedysarum Multijugum Maxim*. [*Leguminosae; Astragali Radix* (Huang Qi)]*, Ligusticum striatum DC*. [*Apiaceae; Chuanxiong Rhizoma* (Chuan Xiong)]*, Pheretima Aspergillum (E.Perrier)* [*Megascolecidae*; *Pheretima* (Di Long)]*, Bombyx Batryticatus* [A desiccated body formed by the 4–5 instar larvae of *Bombyx mori Linnaeus* (family: *Bombycidae*) infected with white *Beauveria bassiana (Bals.) Vuillant*; (Jiang Can)], and can reduce serum tumor necrosis factor (TNF) -α and plasma thromboxane B2 (TXB2) levels and increase plasma 6-Keto-PGF1α (He et al., 2002). Our previous research also found that HCC can protect neurons in the hippocampal CA_2_ region by regulating Fpn expression to balance iron levels after cerebral ischemia. This suggests that imbalance of intracellular iron balance may be a new mechanism of cerebral ischemia (Liao et al., 2015). However, the mechanism of HCC in treating cerebral ischemia is still not fully explained. Systematic pharmacology is an emerging discipline based on the intersection and integration of multidisciplinary technologies such as classic pharmacology, computer technology, bioinformatics, and network pharmacology, which systematically studies the interactions between drugs and the human body at multiple levels, including molecules, cells, organs, and networks (Zeng et al., 2017; Zeng and Yang, 2017; Bao et al., 2019; Yang et al., 2019a; Yang et al., 2019b). Our previous research used systematic pharmacological strategies to reveal the mechanism of Chinese medicine formula in the treatment of complex diseases in the fields of oncology and cardiovascular (Zeng et al., 2017; Zeng and Yang, 2017; Bao et al., 2019; Yang et al., 2019a; Yang et al., 2019b). Therefore, this study hopes to reveal the complex mechanism of HCC through a systematic pharmacology strategy (integrating network pharmacology experimental pharmacology).

## Materials and Methods

### HCC’s Compounds Collection

The components of HCC were collected from the traditional Chinese Medicine (TCM) Database at Taiwan (http://tcm.cmu.edu.tw/zh-tw/) (Chen et al., 2014) and the Traditional Chinese Medicine Systems Pharmacology Database (TcmSPTM, http://tcmspw.com/tcmsp.php) (Ru et al., 2014). The components with oral bioavailability (OB) ≥ 30%, Caco-2 permeability > −0.4 and drug-likeness (DL) ≥ 0.18 were considered as the potential bioactive compounds of HCC (Walters and Murcko, 2002; Ano et al., 2004; Hu et al., 2009; Xu et al., 2012; Ru et al., 2014). Meanwhile, since the application of biological models to predict HCC compounds has limitations (Metodiewa et al., 1997), a large number of references were searched to supplement oral absorbable compounds with pharmacological activity (Shi et al., 2014; Tong et al., 2019). The components of HCC were shown in Table 1.

**TABLE 1:** Components of HCC.

Drug name	Species	Family	Components
*Astragali Radix (Huang Qi)*	*Hedysarum Multijugum Maxim*	*Leguminosae*	1,7-Dihydroxy-3,9-dimethoxy pterocarpene, 3,9-di-O-methylnissolin, 64474–51-7, 64997–52-0, 73340–41-7, 7-O-methylisomucronulatol, astragaloside IV, Bifendate, Calycosin, Calycosin 7-O-glucoside, Formononetin, Hederagenin, Isodalbergin, Isorhamnetin, Jaranol, Kaempferol, Mairin, Ononin, Quercetin
*Chuanxiong Rhizoma (Chuan Xiong)*	*Ligusticum striatum DC.*	*Apiaceae*	Butylidenephthalide, Butylphthalide, Chlorogenic acid, Coniferyl Ferulate, Ferulic acid, Ligustilide, Ligustrazine, Mandenol, Myricanone, Perlolyrine, Senkyunolide A, Senkyunolide H, Senkyunolide I, Senkyunone, Sitosterol, Wallichilide
*Pheretima (Di Long)*	*Pheretima Aspergillum (E.Perrier)*	*Megascolecidae*	4-Guanidino-1-butanol, Cholesteryl ferulate, Guanosine, Hyrcanoside, Xanthinin
*Bombyx Batryticatus (Jiang Can)* ^a^	*-*	*-*	Bassianin, Beauvericin, Ecdysterone, Ergotamine, Lupeol acetate

a *Bombyx Batryticatu* is the desiccated body formed by the 4–5 instar larvae of *Bombyx mori Linnaeus* (family: *Bombycidae*) infected with white *Beauveria bassiana (Bals.) Vuillant*.

### HCC’s Potential Targets Prediction and CIR Genes Collection

The molecular structure was drawn by ChemBioDraw according to their structure in SciFinder (http://scifinder.cas.org) and saved as “mol2” file format. The “mol2” files of HCC’s components were input into PharmMapper (http://lilab-ecust.cn/pharmmapper) for potential targets prediction (Wang X. et al., 2017). OMIM database (http://omim.org/) and Genecards (http://www.genecards.org) were utilized to collect the CIR-related disease genes and targets (Hamosh et al., 2005; Stelzer et al., 2016). The PDB ID number of HCC’s protein target and the name of CIR-related targets were input into UniProt KB (https://www.uniprot.org/uniprot/) to obtain the official gene symbol of each target. (see Supplementary Table S1 and Supplementary Table S2 in Supplementary Material).

### Network Construction and Analysis Methods

The protein-protein interaction (PPI) data of targets were obtained from String 11.0 (http://string-db.org/) (Szklarczyk et al., 2015). The CIR gene, HCC compounds and potential targets, and PPI data were imported into Cytoscape 3.7.1 software (https://cytoscape.org/) for network construction (Bader and Hogue, 2003). The networks were analyzed by the plugin MCODE to obtain cluster. The definition and the methodology of acquisition of clusters were described in our previous work (Zeng et al., 2017; Zeng and Yang, 2017; Bao et al., 2019; Yang et al., 2019a; Yang et al., 2019b), such as “Exploring the Pharmacological Mechanism of Quercetin-Resveratrol Combination for Polycystic Ovary Syndrome: A Systematic Pharmacological Strategy-Based Research” (Yang et al., 2019a) and “Uncovering the Pharmacological Mechanism of *Astragalus* Salvia Compound on Pregnancy-Induced Hypertension Syndrome by a Network Pharmacology Approach” (Zeng et al., 2017).

The HCC targets and CIR genes in the network were input into the Database for Annotation, Visualization and Integrated Discovery (DAVID, https://david-d.ncifcrf.gov, ver. 6.8) for Gene Ontology (GO) enrichment analysis and pathway enrichment analysis (Huang et al., 2009).

### Experimental Materials

#### Instruments and Reagents

Instruments: BX51 optical microscope, Motic Image Advanced 3.0 image analysis system (Olympus, Japan); Tgradient PCR instrument (Biomatra); KD2258 paraffin slicer (Zhejiang Jinhua); LEICA DM LB2 binocular microscope (Leica, Germany); JY3002 electronic balance (Shanghai Precision Scientific Instrument Co., Ltd.); HHS-2 electronic constant temperature stainless steel Water bath (Shanghai Nanyang Instrument Co., Ltd.); S2-93 automatic double pure water distiller (Shanghai Yarong Biochemical Instrument Factory). Eppendorf benchtop cryogenic microcentrifuge (Eppendorf Inc.); high speed benchtop centrifuge (Beckman Inc.); micropipette (Eppendorf Inc.) ultrapure water meter (Millipore Inc.); ultra clean bench (Suzhou Purification Equipment Co., Ltd.); −80°C ultra-low temperature refrigerator (Zhongke Meiling Cryogenic Technology Co., Ltd.). Column: Welch Vltimate XB-C18 (HS), 4.6 x 250 nm. 5 μm; High Performance Liquid Chromatograph: Agilent, Germany angilent1260 (Diode Array Detector).

Reagents: TRIzol was purchased from Invitrogen Inc., United States; RT-PCR kit was purchased from MBI Inc, United States; HIF-1α (batch number: bs-0737R), VEGF (batch number: bs-1957R) and VEGFR (batch number: bs-0565R) antibody were purchased from Beijing Boaosen Biotechnology Co., Ltd.; vWF monoclonal antibody (batch number: F3520), rhodamine red (lot number: 83697), isothiocyanate fluorescent yellow (FITC, batch number: P5282) were purchased from Sigma Inc., United States; BrdU monoclonal antibody (NA61) was purchased from Chemicon, United States; immunohistochemistry kit was purchased from Beijing Boaosen Biotechnology Co., Ltd.; Standards: Ligustrazine Hydrochloride 110817–201608, ferulic acid 110773–201614, astragaloside 110781–201717, all from China Food and Drug Testing Institute.

#### Animal

One hundred and twenty (120) Sprague-Dawley (SD) rat with 7 ∼ 8 week old and body weight 280∼300 g (without limited to male and female) were purchased from Shanghai Xipuer-Bikai Laboratory Animal Co., Ltd. [Animals permit number: SCXK (Shanghai) 2018–0016]. All animals were housed in clean cages and housed under a 12 h light/dark cycle at a temperature of 21–25°C and a humidity of 45–65%. Free drinking, feeding, and adaptive feeding for 1 week. The experiment was approved by the Ethics Committee of Hunan University of Chinese Medicine (Changsha, China) (Approval No: 201404163). Animal experiments are strictly in accordance with the ethics committee guidelines and laboratory animal care and use guidelines.

#### Preparation of HCC Extract

*Hedysarum Multijugum Maxim*. (Specimen number: 2014062101)*, Chuanxiong Rhizoma* (Specimen number: 2014062410)*, Pheretima* (Specimen number: 2014062205)*, Bombyx Batryticatus* (Specimen number: 20140622307), with ratio 4:1:1.5:1.5, was purchased from the Chinese Pharmacy of the First Affiliated Hospital of Hunan University of Chinese Medicine. The herbs were verified by Professor Bing Dai. The HCC extract was prepared by the Department of Pharmacy, the First Affiliated Hospital of Hunan University of Chinese Medicine. The specific experimental procedure refers to our previous study (Chen, 2005). When in use, the HCC dry extract and physiological saline are formulated into an HCC solution.

#### High Performance Liquid Chromatography

Three representative compounds in HCC were identified by high performance liquid chromatography (HPLC) for quality control: astragaloside IV, ferulic acid, and ligustrazine. Conditions: Ultimate XB-C18 column (5 μm, 4.6 × 250 mm), A: acetonitrile; B: 0.2% phosphoric acid-water; gradient elution flow rate: 1 ml/min; Detection wavelength: 198, 201, 280, 290, 315, 320 nm; injection volume: 10 μl.

The HPLC of HCC is shown in Supplementary Figure S1. The main compounds of HCC were quantified: astragaloside IV 13.72 mg/200g, ferulic acid 2.52 mg/200g, ligustrazine 0.36 mg/200g.

### Experimental Methods

#### Animal Grouping, Model Preparation and Intervention Methods

One hundred and twenty (120) rats were randomly divided into normal group (*n* = 12), sham operation group (*n* = 12), CIR model group (*n* = 48) and HCC group (*n* = 48). The model group and the HCC group were divided into four subgroups (1, 3, 5, and 7 days after reperfusion) with 12 rats in each group. All animals were trained for 3 days with reference (Bederson et al., 1986; Huang et al., 2009) before modeling. The specific method is: suspending the tail of the rat about 1 m from the ground, and observing the flexion of the forelimb. The cages where the animals are kept are marked with letters, and the drugs are also marked with letters, and they are kept by a third person before the end of the experiment. The animal experiment operator, data collector, and indicator tester do not know the group and intervention drugs.

Scoring criteria: 0 points: Rat forelimbs extended to the ground, no other symptoms; 1) point: The forelimb of the injured hemisphere of the rat suffered from flexion, and its posture changed from slight lumbar flexion and a certain degree of adduction to the shoulder of the elbow, to complete flexion of the waist and elbow and internal rotation of the shoulder; 2) points: Put the rat on a piece of soft plastic paper, lift the tail, apply a soft force on the shoulder, and the resistance of the rat to the external force which causes it to slide toward the diseased side is weakened; 3) points: The rat has a clear consciousness, making a rear-end movement to the right or falling to the right; 4) points: accompanied by disturbance of consciousness, no spontaneous activity; 5) points: death. The entire test process takes 3–5 min, and the rats with scores of 1–3 were enrolled. When the rat died and the sample size was insufficient, random replacement was performed. The left middle cerebral artery occlusion (MCAO) model was prepared by referring to Longa et al. (1989). Rats were fasted before operation and anesthetized. The body temperature was maintained by a constant temperature circulating water system. The rat had a median neck incision, exposed the left external carotid artery and its branches (occipital artery and superior thyroid artery) and the common carotid artery bifurcation. The occipital artery and the superior thyroid artery were ligated, and the carotid artery bifurcation was cut at the bifurcation of the common carotid artery and a smooth nylon thread (1.85 ± 1.5) cm with a diameter of 0.26 mm was inserted into the internal carotid artery. Stop sending the line when the resistance is felt, and record the time. Two hours after the middle cerebral artery was blocked, the suture was withdrawn to restore perfusion. The sham operation group only inserted the fishing line about 1 cm, and the others were the same as the model group. The success rate of modeling is about 80%, and the failure model is randomly replaced. After the model is prepared, the animals are kept in cages.

Administration method: HCC group: the dose given to rats was calculated according to the animal body surface area dose conversion algorithm, the formula is D2 = D1 × R2 ÷ R1 (D2 is the desired dose, D1 is the known dose, R1 is the corresponding known value, and R2 is the ratio of the surface area of the corresponding animal body). The daily dose for rats (0.72 g/ml, 1 ml/100 g) is equivalent to twice the dose for adults. It was calculated from the ratio of 80 g of the daily dose of a 70 kg adult to the body surface area of 300 g rat. The first dose was given 2 h after waking anesthesia. The HCC group was intragastrically administered with HCC extract, and the rest were given the same amount of physiological saline, and were given once a day for 7 days.

#### Specimen Collection and Section Preparation

One (1) hour after the last administration, the rats were anesthetized with 1% sodium pentobarbital (2.75 ml/kg, intraperitoneal injection), and then sacrificed by cervical dislocation. The brain tissue was then quickly collected. The specimen was divided into two parts, one was rinsed with 1‰ DEPC water and immediately placed in liquid nitrogen; the other was fixed in 4% paraformaldehyde to make a 5 μm thick paraffin section for HE staining, immunohistochemical staining and BrdU/vWF fluorescence double labeling.

### Detection Indicators and Methods

#### Neurological Deficit Score

Neurological deficits were assessed by a five-point, four-point scoring method by Longa et al. (1989). 0 points: no symptoms of neurological deficits; 1) point: When the rat is suspended from the tail, the contralateral forelimb of the lesion is flexed and raised, the shoulder is adducted, and the elbow is straight; 2) points: rotate to the opposite side of the lesion; 3) points: fall to the opposite side of the lesion; 4) points: no spontaneous activity and decreased level of consciousness. The score was performed at 1, 3, 5, and 7 days after surgery. Rats with a score of 1–3 were selected for inclusion in the results.

#### Volume of Cerebral Infarction

The volume of cerebral infarction was assessed by TTC staining. The brain tissues were and placed in a refrigerator at −20°C for 10 min. Then the brain tissue was deceived and placed in a 1% TTC solution, strictly protected from light, incubated at 37°C for 30 min, and then placed in 10% formaldehyde for 3 h. The stain-free area is infarct tissue. The area of infarct size was calculated by multimedia color image analysis system. The infarct volume was calculated according to formula V = S × D (S is the infarct area of each slice and D is the slice thickness). The infarct size of each brain slice was measured on a computer by area measurement software, and the sum of the infarct volumes of all brain slices was the total volume of the infarct.

#### Microvessel Density Detection

Microvessels were observed by immunohistochemical methods, and microvessel density (MVD) counts were performed according to Weidner et al. (1991). The test was carried out according to the instructions of the immunohistochemistry kit. vWF is labeled as a brownish-yellow particle in the cytoplasm of vascular endothelial cells, and any endothelial cell or endothelial cell cluster that is stained brown by the vWF antibody is considered to be a blood vessel count. Using the MIAS medical image analysis system, five sections were taken at each time point, and each section was selected for 4 high-magnification (× 400) fields around the same cortical ischemic area for microvessel counting. Referring to the Weidener counting method, the number of microvessels per 1 mm^2^ was calculated and then averaged. The results of 15 slices per group at each time point were obtained.

#### Immunofluorescence Staining for Expression of BrdU and vWF

After Brdu is dissolved in normal saline, the dose is determined at 100 mg/kg/day, and intraperitoneal injection is performed. The sections of brain tissue were immersed in 3% H2O2 deionized water for 10 min; washed with PBS for 5 min × 3 times; immersed in 2 mol/L HCl at 37°C for 15 min. Then, the sections were wash with PBS for 5 min × 3 times; 5% goat serum was blocked at room temperature for 30 min, and the liquid was aspirated. After that, BrdU monoclonal antibody (1: 100) 10 μl were added and incubated in 37°C water bath. Then, FITC (luminescence wavelength 520∼530 nm, yellow-green light) staining was performed, 37°C water bath for 30 min, after washing, the sections were blocked with 5% goat serum at room temperature for 30 min vWF antibody (1: 100) 10 μl were added and incubated in37 °C water bath for 3 h. Rhodamine (light emission wavelength 570∼590 nm, red light) staining was performed, 37°C water bath for 30 min. Finally, the slices were packaged with glycerin and observed with an OLYMPUS BX51 fluorescence microscope by the corresponding color filters at 520 and 580 nm, respectively. FITC is green and rhodamine is red. The image is taken in Key Lab of Hunan Province for Prevention and Treatment of Cardio-cerebral Diseases with Integrated Traditional Chinese and Western Medicine, Hunan University of Chinese Medicine, and the images obtained under the two excitation lights are superimposed to obtain a yellow signal as a common signal.

#### Detection of HIF-1α, VEGFA and VEGFR mRNA Levels

The total RNA was extracted from the infarcted side of the brain by the guanidinium isothiocyanate method. The mRNA expression was determined by Reverse Transcription-Polymerase Chain Reaction (RT-PCR).

PCR product analysis: 5 μl PCR amplification products were analyzed on 2.5% agarose gel electrophoresis (voltage 50 V, 30–45 m, ethidium bromide staining). The electrophoresis strips were taken under UV light and the negatives were scanned with a laser density scanner. The expression levels of HIF-1α, VEGF and VEGFR mRNA were calculated using the following formula: Relative product content = HIF-1α (VEGF, VEGFR) amplification product optical density value/GAPDH amplification product optical density value × 100%. The Primers were designed with reference to the computer gene library nucleotide sequence data, and were synthesized by Shanghai Shenggong Bioengineering Co., Ltd. (Table 2).

**TABLE 2:** Primer design.

Gene primer sequence	Product size	/bp
VEGF-A	F: 5'-CGCCAAGCCCGGAAGATTAG-3'R: 5'-CCA​GGG​ATG​GGT​TTG​TCG​TG-3'	392
VEGFR2	F: 5'-CTGTGCTGTTTCCTACCCTAATC-3'R: 5'-CTT​TAC​CGT​CGC​CAC​TTG​AC-3'	275
HIF-1α	F: 5'-TACTGATTGCATCTCCACCTTCTAC-3'R: 5'-CTG​CTC​CAT​TCC​ATC​CTG​TTC-3'	210
GAPDH	F: 5'-AACTCCCTCAAGATTGTCAG-3R: 5'-GGG​AGT​TGC​TTG​AAG​TCA​CA-3'	448

#### Detection of HIF-1α, VEGFA and VEGFR Protein by Immunohistochemistry

The brain tissue was embedded by paraffin, sliced (5 μm), conventional dewaxed. Then it was soaked in 3% hydrogen peroxide for 10 min at room temperature and washed twice with distilled water. After heat repairing the antigen, the primary antibody (rabbit anti-VEGF 1:300; rabbit anti-VEGFR2 1:200; rabbit anti-HIF-1α1:200) and the biotinylated secondary antibody were added sequentially. Then the horseradish enzyme-labeled streptavidin solution was added and incubated in 20∼37°C for 20 min. The color was developed by DAB at room temperature, and the reaction time was controlled under the microscope (5–30 min). Finally, the slices were lightly counterstained with hematoxylin, dehydrated, and transparent, and sealed with a neutral gum. The expression of HIF-1α, VEGFA and VEGFR was observed under a microscope.

### Statistical Analysis

The data were processed by SPSS 19.0 statistical software, and the measurement data were expressed as mean ± standard deviation. Variance homogeneity tests were performed for comparison between groups. If the variances are homogeneous, multiple comparisons using a completely random design analysis of variance or a grouped t test are used for data processing. If the variances are not homogeneous, the rank sum test is used.

## Results and Discussion

### Potential Targets of HCC and CIR Genes

**FIGURE 1:**
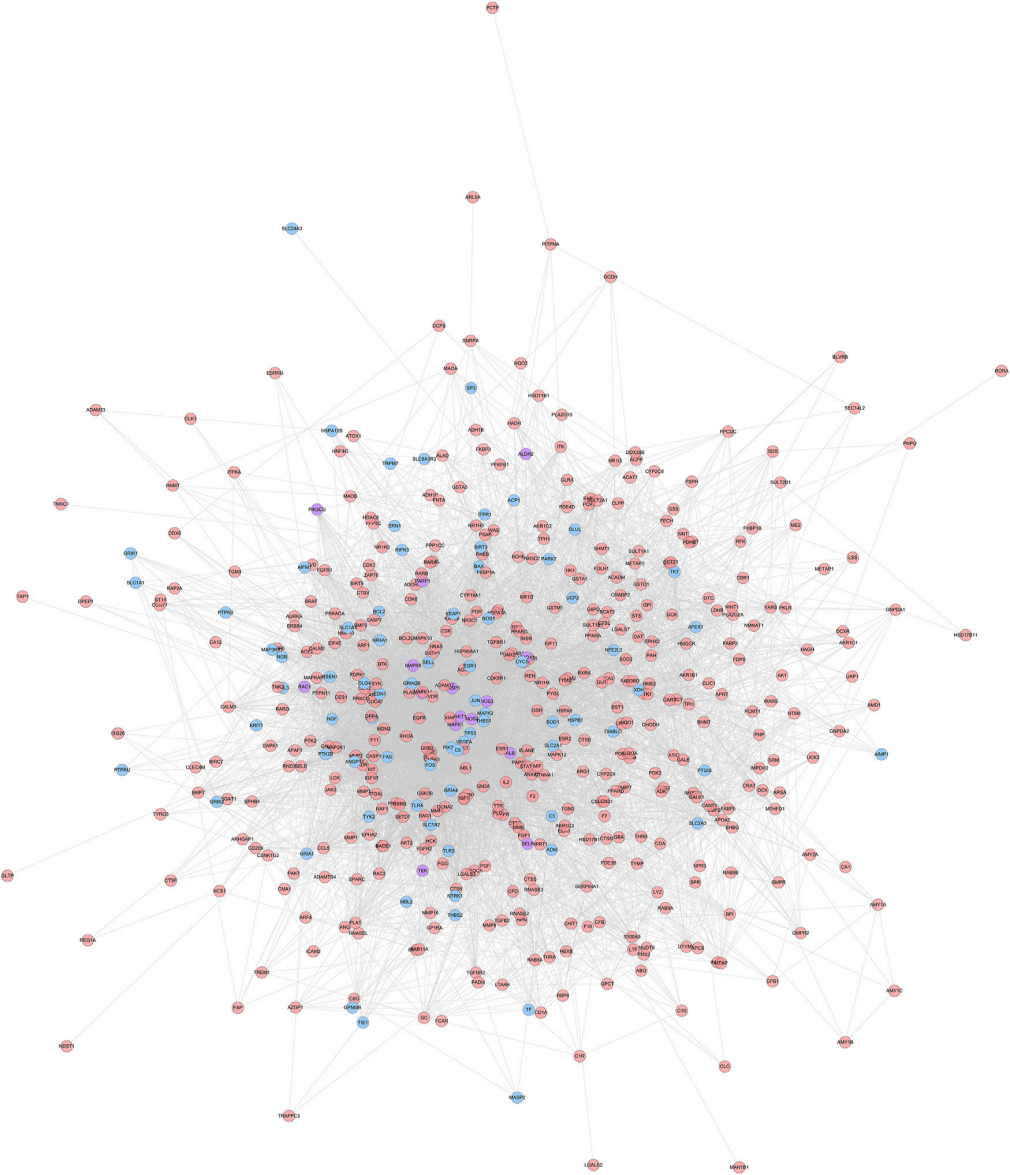
HCC-CIR PPI Network (Blue, pink, purple circles stand for CIR genes, HCC targets and HCC-CIR targets, respectively).

**FIGURE 2:**
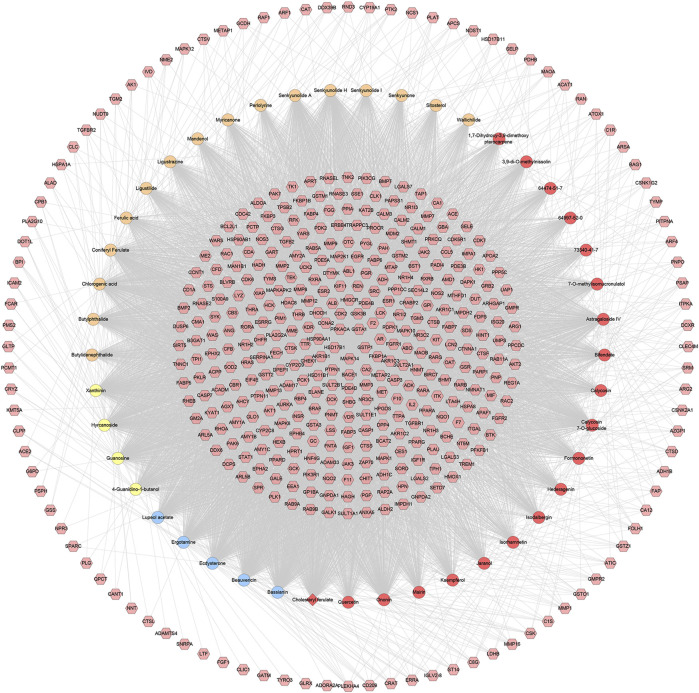
Potential compounds-potential targets network of HCC (The red circle stands for compound of *Hedysarum Multijugum Maxim.*; the blue circle stands for compound of *Bombyx Batryticatus*; the yellow circle stands for compound of *Pheretima*; the orange circle stands for compound of *Chuanxiong Rhizoma*. The red diamond stands for the common compound of *Pheretima* and *Bombyx Batryticatus*. The pink hexagon stands for potential targets.).

Ninety CIR-related genes were obtained from GeneCards and OMIM database (see Supplementary Table S2). After the potential target prediction, totally 440 potential targets were obtained. The relationship among potential compounds and potential targets was shown in Figure 1. This network consists of 440 compound targets, 45 potential compounds and 8,759 edges. The targets near the center are regulated by more compounds than ones in the peripheral. For example, the targets in the center are: AR (45 edges), BACE1 (45 edges), CA2 (45 edges), CDK2 (45 edges), F2 (45 edges), FKBP1A (45 edges), GSK3B (45 edges), GSTA1 (45 edges), GSTP1 (45 edges), HSD17B1 (45 edges), MAPK14 (45 edges), PRKACA (45 edges), AKR1B1 (44 edges), CCNA2 (44 edges), HSP90AA1 (44 edges), MMP3 (44 edges), PDE4D (44 edges), PTPN1 (44 edges), AKR1C3 (43 edges), FGFR1 (43 edges), LCK (43 edges), METAP2 (43 edges), PDPK1 (43 edges); the targets in the peripheral (ADAMTS4, ADORA2A, CLIC1, FGF1, GATM, GLRX, LTF) are regulated only by one compound. The compounds in herbs can regulate multiple targets. The top five compounds in each herb are: *Hedysarum Multijugum Maxim.*: Astragaloside IV (298 edges), Calycosin 7-O-glucoside (295 edges), Ononin (294 edges), Bifendate (162 edges), Isorhamnetin (151 edges); *Chuanxiong Rhizoma*: Senkyunolide H (297 edges), Coniferyl Ferulate (295 edges), Chlorogenic acid (295 edges), Senkyunolide A (219 edges), Butylphthalide (218 edges); *Pheretima*: Hyrcanoside (298 edges), Cholesteryl ferulate (295 edges), Xanthinin (288 edges), Guanosine (283 edges), 4-Guanidino-1-butanol (255 edges); *Bombyx Batryticatus*: Ergotamine (297 edges), Ecdysterone (297 edges), Beauvericin (296 edges), Bassianin (295 edges), Lupeol acetate (228 edges) (Figure 2).

### HCC-CIR PPI Network Analysis

#### HCC-CIR PPI Network

HCC potential targets, CIR genes and their PPI data were input into Cytoscape to construct HCC-CIR PPI network. This network contains 502 nodes 75 CIR gene nodes, 412 HCC target nodes and 15 HCC-CIR targets nodes) and 7,762 edges (Figure 1) The top 20 targets of degree are selected and divided into three categories: 1) CIR targets: TP53 (189 edges), VEGFA (166 edges), MAPK3 (164 edges), JUN (133 edges), CYCS (109 edges); 2) HCC genes: EGFR (155 edges), HSP90AA1 (135 edges), HRAS (127 edges), IGF1 (126 edges), CAT (123 edges), MMP9 (122 edges), ESR1 (116 edges), RHOA (104 edges), MAPK14 (102 edges); 3) HCC-CIR targets: ALB (215 edges), AKT1 (205 edges), MAPK1 (156 edges), SRC (143 edges), CASP3 (141 edges), MAPK8 (137 edges).

#### Clusters of HCC-CIR PPI Network

**FIGURE 3:**
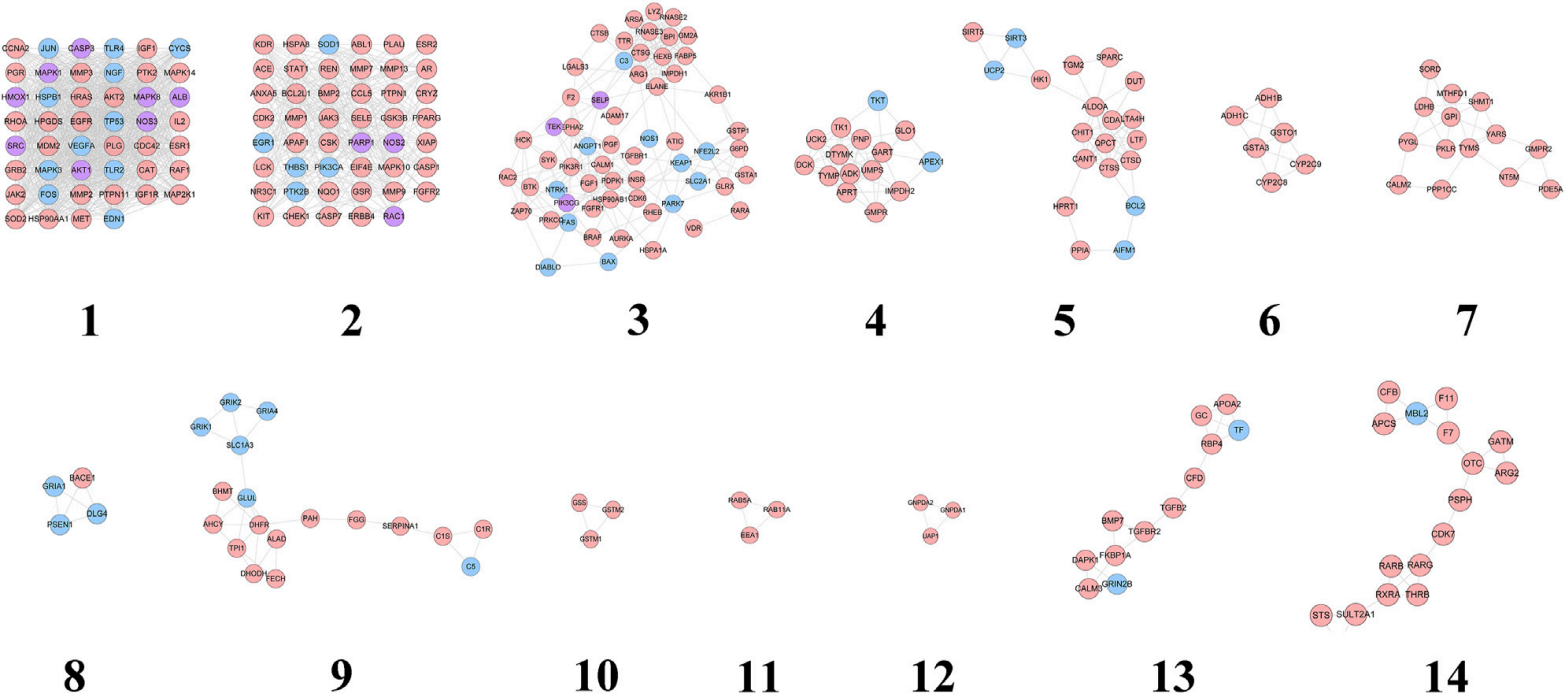
Cluster of HCC-CIR PPI Network (Blue, pink, purple circles stand for CIR genes, HCC targets and HCC-CIR targets, respectively).

The HCC-CIR PPI network was analyzed by MCODE, and returns 14 clusters (Table 3 and Figure 3).

**TABLE 3:** Cluster of HCC-CIR PPI Network.

Cluster	Score	Nodes	Edges	Genes and targets
1	35.244	46	793	MAPK14, SOD2, MAPK8, MDM2, SRC, MET, PLG, EGFR, TP53, CASP3, CAT, HMOX1, MMP2, ESR1, MMP3, HSPB1, CCNA2, HPGDS, FOS, TLR4, MAPK3, HRAS, JUN, AKT1, AKT2, CDC42, PTK2, ALB, HSP90AA1, PTPN11, CYCS, NOS3, IGF1, IGF1R, IL2, RAF1, EDN1, JAK2, RHOA, NGF, TLR2, VEGFA, GRB2, PGR, MAP2K1, MAPK1
2	11	47	253	MAPK10, ABL1, ACE, PLAU, THBS1, STAT1, EIF4E, MMP1, CASP1, MMP13, CASP7, PPARG, ERBB4, ESR2, CCL5, MMP7, MMP9, CDK2, PTK2B, PTPN1, NOS2, HSPA8, NQO1, CHEK1, RAC1, FGFR2, ANXA5, APAF1, NR3C1, AR, XIAP, CRYZ, CSK, JAK3, REN, EGR1, KDR, SOD1, KIT, PARP1, LCK, PIK3CA, BCL2L1, SELE, BMP2, GSK3B, GSR
3	7.932	60	234	BRAF, SLC2A1, GSTA1, BTK, NOS1, GSTP1, ANGPT1, CALM1, HCK, HEXB, PRKCQ, FAS, CDK6, HSP90AB1, HSPA1A, C3, RAC2, IMPDH1, INSR, RARA, DIABLO, PARK7, RHEB, CTSB, RNASE2, RNASE3, CTSG, LGALS3, LYZ, SELP, ADAM17, ELANE, EPHA2, SYK, TEK, AKR1B1, TGFBR1, F2, FABP5, TTR, FGF1, FGFR1, VDR, G6PD, ARG1, ZAP70, ARSA, BAX, ATIC, NTRK1, AURKA, NFE2L2, GLRX, GM2A, PDPK1, KEAP1, PGF, PIK3CG, BPI, PIK3R1
4	7.714	15	54	TKT, TYMP, IMPDH2, UCK2, DTYMK, UMPS, APRT, PNP, APEX1, GART, ADK, GLO1, GMPR, TK1, DCK
5	5.579	20	53	SIRT5, CHIT1, SPARC, DUT, CANT1, BCL2, HK1, PPIA, CTSD, AIFM1, HPRT1, TGM2, CTSS, CDA, LTA4H, LTF, SIRT3, ALDOA, UCP2, QPCT
6	4.4	6	11	CYP2C8, GSTA3, CYP2C9, ADH1B, GSTO1, ADH1C
7	3.846	14	25	TYMS, PKLR, SORD, NT5M, CALM2, YARS, PPP1CC, LDHB, MTHFD1, PDE5A, GMPR2, GPI, PYGL, SHMT1
8	3.333	4	5	PSEN1, GRIA1, BACE1, DLG4
9	3.176	18	27	FECH, DHFR, GRIK2, DHODH, C1R, C1S, C5, FGG, PAH, AHCY, GLUL, SLC1A3, ALAD, BHMT, GRIK1, SERPINA1, TPI1, GRIA4
10	3	3	3	GSTM2, GSS, GSTM1
11	3	3	3	EEA1, RAB11A, RAB5A
12	3	3	3	UAP1, GNPDA1, GNPDA2
13	2.909	12	16	GC, GRIN2B, TGFB2, TGFBR2, FKBP1A, APOA2, RBP4, CALM3, DAPK1, TF, BMP7, CFD
14	2.875	17	23	MBL2, APCS, RARB, RARG, STS, OTC, ARG2, SULT2A1, GATM, F11, RXRA, AKR1C3, F7, THRB, PSPH, CDK7, CFB

These genes in each cluster were input into DAVID database to undergo GO enrichment analysis. After that, several biological processes were obtained. Cluster 1 is associated with negative regulation of neuronal apoptosis, nitric oxide production and metabolism, angiogenesis, hypoxia induction, neurotrophicity, platelet activation, and CIR-related signaling pathways (such as NF-kB signaling pathway). Cluster 2 is related to cell proliferation, hypoxia-induced, cell migration, and CIR-related signaling pathways (ERK1/2 signaling and PI3K signaling, EGF receptor signaling, and VEGF receptor signaling pathways). Cluster 3 mainly involves inflammation, platelet activation, nerve cell survival, angiogenesis, oxidative stress, neuronal axonal injury, hypoxia-induced stress, blood coagulation, calcium homeostasis. Cluster 4 is associated with nucleic acid metabolism. Cluster 5 is related to oxidative stress, neuronal apoptosis, ischemia induction, cytokines and NF-κB signaling pathway. Cluster 8 mainly includes the biological processes of synaptic transmission. Cluster 9 is associated with chemical synapses and neurotransmitters. Cluster 13 mainly involves angiogenesis. Cluster 6, 7, 10, 11, 12, 14 do not return CIR-related biological processes (see Supplementary Table S3).

**FIGURE 4:**
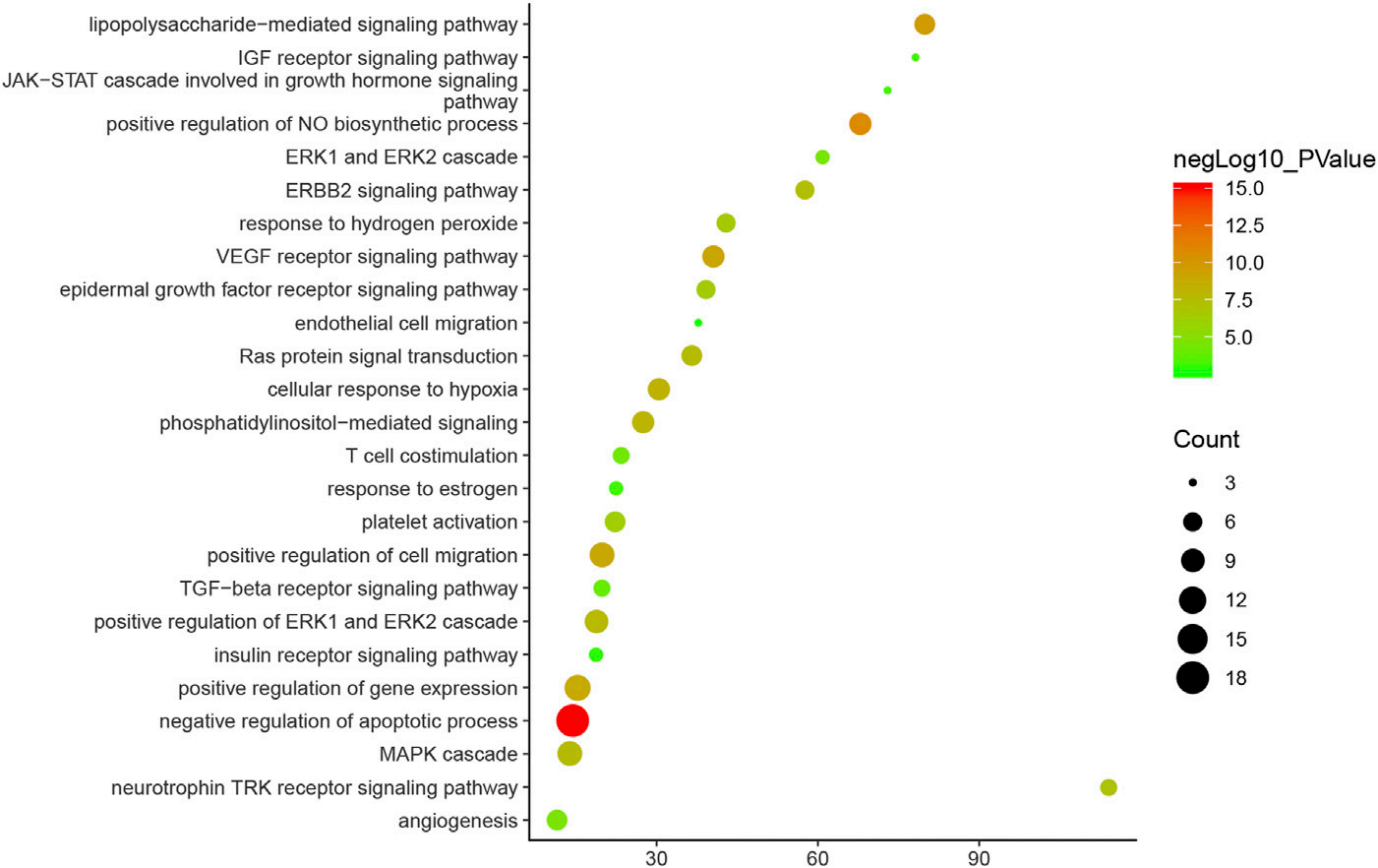
Bubble chart of biological processes (*X*-axis stands for fold enrichment).

Since cluster 1 is the most important one, it is used as an example to show its main biological processes on bubble chart (Figure 4).

#### Pathway of HCC-CIR PPI Network

**FIGURE 5:**
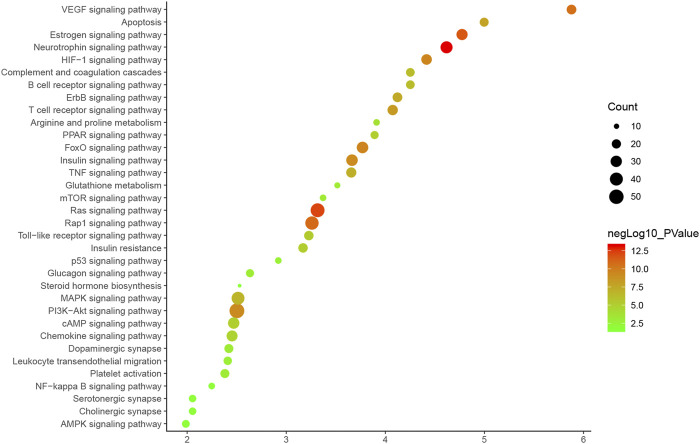
Bubble Chart of Signaling Pathway (*X*-axis stands for fold enrichment).

Thirty-four CIR signaling pathways are obtained. The signaling pathway is arranged according to the degree of enrichment (based on *p*-value) and count, and the Neurotrophin signaling pathway is found to have the highest enrichment and contained 34 targets (*p*-value = 7.15*10 ^-14; Count = 34). According to the sorting, the other signaling pathways (top 10) are: Ras signaling pathway (*p*-value = 6.74*10 ^-13; Count = 46), Estrogen signaling pathway (*p*-value = 2.82*10 ^-12; Count = 29), Rap1 signaling pathway (*p*-value = 1.49*10 ^-11; Count = 42), VEGF signaling pathway (*p*-value = 2.54*10 ^-11; Count = 22), FoxO signaling pathway (*p*-value = 2.86*10 ^-10; Count = 31), HIF-1 signaling pathway (*p*-value = 3.04*10 ^-10; Count = 26), Insulin signaling pathway (*p*-value = 6.15*10 ^-10; Count = 31), PI3K-Akt signaling pathway (*p*-value = 6.46*10 ^-10; Count = 53), T cell receptor signaling pathway (*p*-value = 4.18*10^-09; Count = 25) (See Figure 5). (Supplementary Table S4).

Through chemical informatics technology, combined with the prediction and analysis of active ingredients and potential targets, protein interaction analysis and gene annotation enrichment analysis, we systematically explored the pharmacological substance basis and potential biological mechanism of HCC for CIR. Estrogen signaling pathways are involved in CIR’s blood-brain barrier, neuroprotection, and oxidative stress (Yang et al., 2005; Wiklund et al., 2012). VEGF signaling pathway is involved in angiogenesis after CIR (Sun et al., 2015; Ho et al., 2017). FoxO signaling pathway is involved in oxidative stress in CIR (Yoo et al., 2012; Zhou et al., 2019). HIF-1 signaling pathway is involved in the induction of hypoxia after CIR (Singh et al., 2012). Insulin signaling pathways are involved in vascular endothelial apoptosis (White et al., 2000). The PI3K-Akt signaling pathway and T cell receptor signaling pathway are involved in cell damage of CIR (Huang et al., 2007; Zhou et al., 2015). HCC may play a therapeutic role by regulating these CIR-related signaling pathways.

Recent studies have also shown that *Astragalus* injection can promote cerebral vascular regeneration in CIR rats, the mechanism of which may be that *Astragalus* injection activates the HIF-1α/VEGF signaling pathway (Wu et al., 2016). Astragaloside IV can promote the proliferation and differentiation of neural stem cells in the hippocampus, inhibit the activation of astrocytes and microglia after CIR in rats and the release of inflammatory factors, and protect the integrity of the blood-brain barrier (Qu et al., 2009; Li et al., 2017; Huang et al., 2018; Li et al., 2018). It can also increase the expression of brain-derived neurotrophic factor (BDNF), vascular endothelial growth factor (VEGF) and VEGF receptor 2 (VEGFR2) after CIR, promote the formation of new blood vessels, improve the survival environment of nerve cells, and inhibit apoptosis or necrosis of ischemic hypoxic neurons (Yang L. et al., 2019). Astragaloside IV may protect CIR by reducing catalase (CAT), superoxide dismutase (SOD), glutathione peroxidase (GSH-Px) activity, malondialdehyde (MDA) content in brain tissue, lactate dehydrogenase (LDH) and creatine kinase (CK) content in serum, and reduce the expression of NF-κB protein in the brain due to CIR (Shao et al., 2014). This also indirectly confirms the reliability of our reverse pharmacophore docking technology in chemical informatics. Ligustrazine, as the main compound of Chuanxiong Rhizoma, can alleviate the energy metabolism disorder of CIR (Wang Z. et al., 2017), reduce excitatory amino acid toxicity, inhibit apoptosis and the synthesis of inflammatory cells and pro-inflammatory cytokines IL-1 and TNF-α, and fight against inflammatory damage caused by IL-1 and TNF-α (Wang A. et al., 2017; Zhao et al., 2018). Ligustrazine can also induce adrenocortical hormone production, control multiple links of the inflammatory response, increase SOD activity in the brain, reduce NOS expression, and affect nitric oxide (NO) content (Peng et al., 1996; Zhou et al., 2000; Wang A. et al., 2017).

In addition, this study also found that there may be a potential synergy between HCC active compounds. For example, astragaloside IV and ferulic acid can jointly regulate: AURKA, CBS, HPGDS, RAC2, DCK, PDE4B, AMY1A, OTC, CCL5, ANG, AGXT, AMD1, ELANE, PDE4D, PTPN1, MMP3, RARG, UCK2, AMY2A, REN, PDE5A, HMGCR, SHMT1, F7, ADK, GLO1, LYZ, RHOA, EPHA2, TPI1, et al. Ferulic acid and ligustrazine can jointly regulate: HSP90AA1, PDE4D, GPI, MME, FKBP1A, AR, DCK, ABO, CCNA2, AMY1B, GSTP1, EGFR, F2, PPP1CC, PDE4B, SDS, PDPK1, GLO1, LCK, CA2, PRKACA, AMY1C, PIM1, REN, HDAC8, GCK, MMP3, MAPK10, CYP2C9, GSTT2, et al. Recent research has confirmed some of our results. Wang (2012) found that electrospun fibers carrying astragaloside IV and ferulic acid can promote angiogenesis. Gong et al. Found that astragaloside IV and ferulic acid can improve blood lipids, protect the cardiovascular system, and have anti-atherosclerotic effects in New Zealand rabbits (Gong and Huang, 2017). In terms of angiogenesis, ligustrazine combined with astragaloside IV can promote angiogenesis of chick embryo chorionic urea capsule (Zhang et al., 2010). Yang et al. Found that the combination of Hedysarum Multijugum Maxim. And Chuanxiong Rhizoma can significantly improve the morphology of hypoxic rat brain microvascular endothelial cells (RBMVECs), effectively enhance the activity of SOD, inhibit the G1/S phase arrest of RBMVECs induced by hypoxia, significantly reduce cell apoptosis, and reduce the expression of caspase-3 and caspase-8 genes (Yang et al., 2015). The results of their orthogonal experiments showed that the preferred combination was ligustilide 5 μg/ml, ligustrazine 10 μg/ml, ferulic acid 20 μg/ml, calycosin 10 μg/ml, astragaloside IV 10 μg/ml (Yang et al., 2015). These have brought huge opportunities for the research and development of new drugs in the future.

### The Effect of HCC on The Score of Neurological Deficit in Rats

The scores of neurological deficits of rats in the sham operation group were 0 points 1 day to 7 days after reperfusion. At 1 d, the scores of neurological deficits in HCC group and CIR model group were higher than those in sham operation group (*p* < 0.05), but the difference between the two groups had no statistical significance. At 3 d, the scores of the neurological deficits in the HCC group were lower than those in the CIR model group (*p* < 0.05). At 5 d and 7 d, the scores of the HCC group and CIR model group decreased, but the scores of CIR model group was still higher than that of sham operation group (*p* < 0.05). The results are shown in Table 4.

**TABLE 4:** Effect of HCC on the score of neurological deficits in rats after CIR at different time points (*n* = 5, x ± s).

Group	1 day	3 days	5 days	7 days
Normal	0	0	0	0
Sham operation	0	0	0	0
CIR model	1.6 ± 0.54^a^	1.6 ± 1.67^a^	0.75 ± 0.5^a^	0.6 ± 0.55^a^
HCC	1.5 ± 1.00^a^	0.5 ± 0.35^b^	0.4 ± 0.54	0.4 ± 0.54

a compared with the Sham operation group, *p* < 0.05.

b compared with the CIR model group, *p* < 0.05.

### Pathological Changes

**FIGURE 6:**
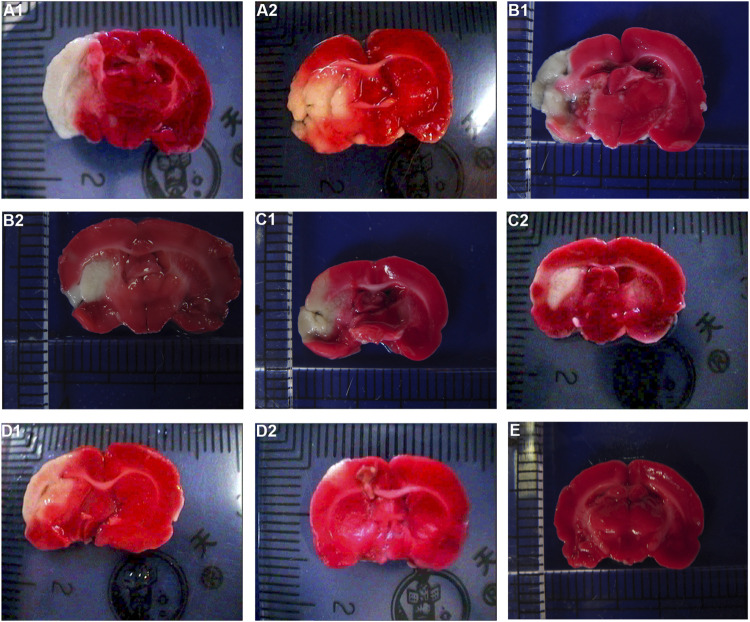
Effect of HCC on cerebral infarction volume in rats at different time after CIR (TCC staining. **(A1)**: Model group 1 day; **(A2)**: HCC group 1 day; **(B1)**: Model group 3 days; **(B2)**: HCC group 3 days; **(C1)**: Model group 5 days; **(C2)**: HCC group 5 days; **(D1)**: Model group 7 days; **(D2)**: HCC group 7 days. **(E)**: Sham operation group).

For the volume of cerebral infarction in rats: after staining with TTC, the uninfarcted area was red and the infarcted area was white. No significant cerebral infarction was seen in the sham operation group. There was no significant difference in cerebral infarction volume between HCC group and model group at 1 d (*p* > 0.05). Compared with model group in the same phase, cerebral infarction volume of the HCC group decreased from 3 days to 7 days (*p* < 0.05). See Figure 6 and Table 5.

**TABLE 5:** Effect of HCC on cerebral infarction volume at different time points after CIR (*n* = 5, mm 3, x ± s).

Group	1 day	3 days	5 days	7 days
Normal	0	0	0	0
Sham operation	0	0	0	0
CIR model	23.59 ± 10.42^a^	26.52 ± 18.03^a^ ^,^ ^b^	25.91 ± 13.61^a^ ^,^ ^b^	22.47 ± 8.70^a^ ^,^ ^b^
HCC	22.14 ± 12.75^a^	12.54 ± 9.04	12.40 ± 4.56	10.19 ± 7.2

a compared with the Sham operation group, *p* < 0.05.

b compared with the HCC group, *p* < 0.05.

**FIGURE 7:**
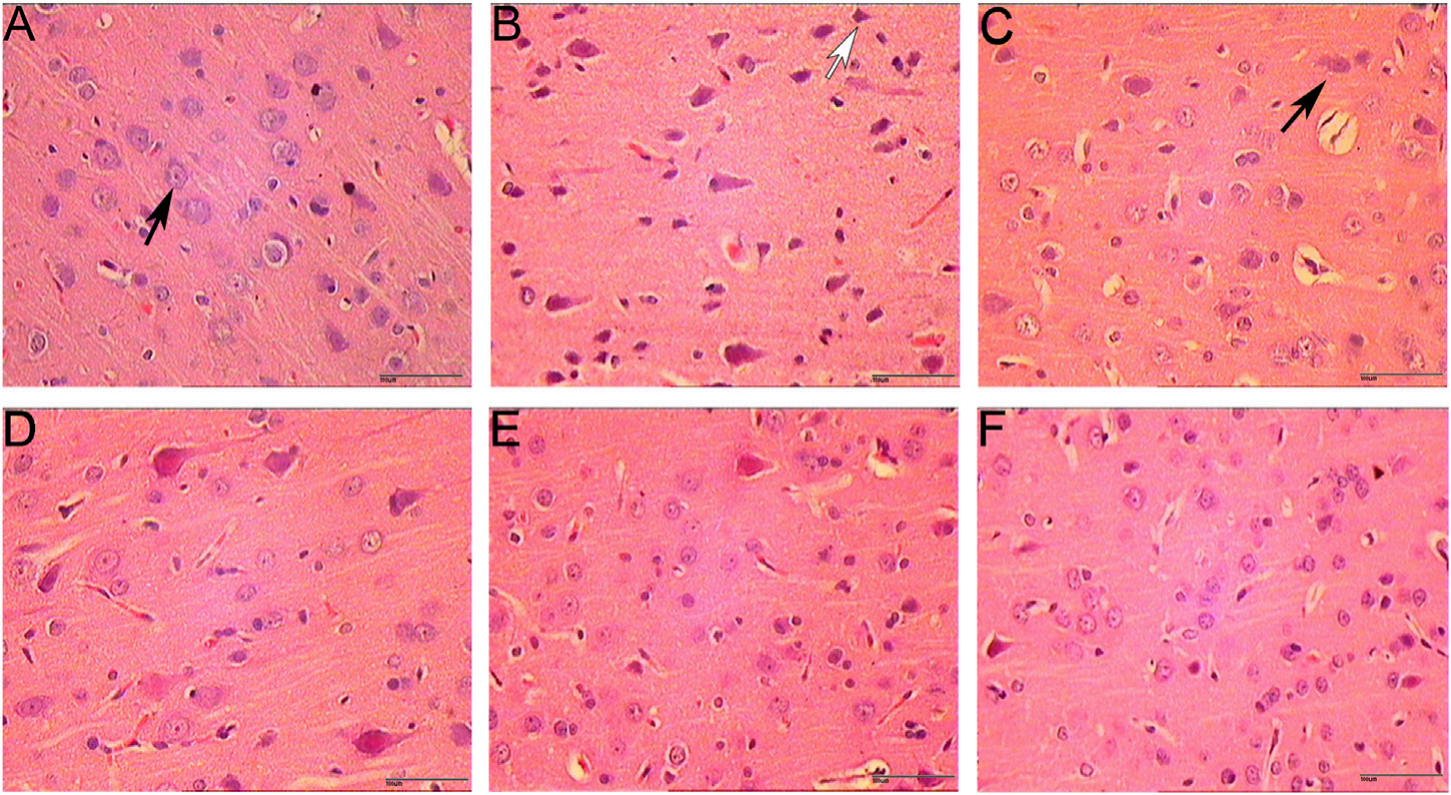
Pathological changes in cerebral cortex (HE staining. 400 x **(A)**: Sham operation group 1 day; **(B)**: Model group; **(C)**: HCC group 1 day; **(D)**: HCC group 3 days; **(E)**: HCC group 5 days; **(F)**: HCC group 7 days; Black arrows indicate normal cells; white arrows indicate cells with pyknotic nucleus.).

**FIGURE 8:**
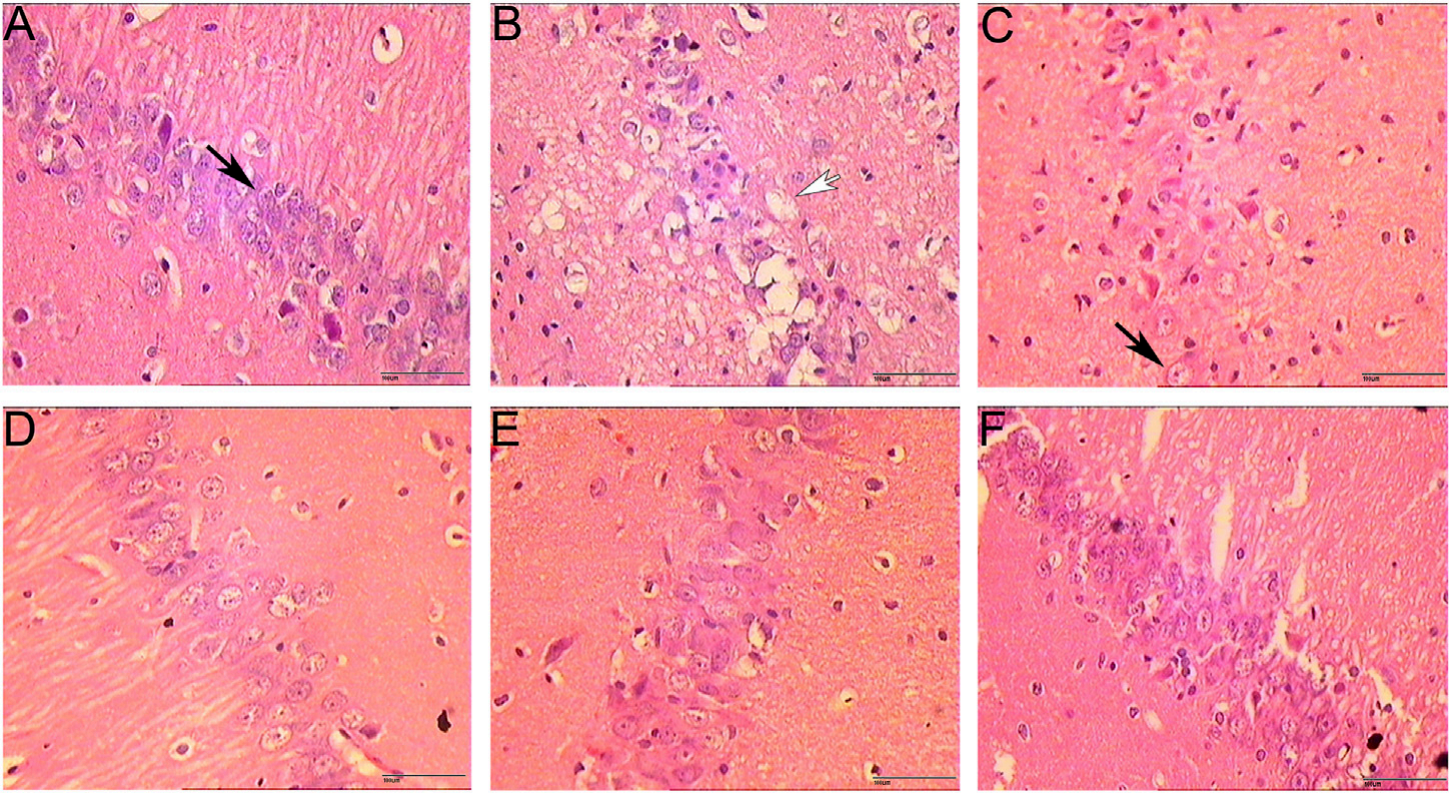
Pathological changes in hippocampus (HE staining. 400 x **(A)**: Sham operation group 1 day; **(B)**: Model group; **(C)**: HCC group 1 day; **(D)**: HCC group 3 days; **(E)**: HCC group 5 days; **(F)**: HCC group 7 days; Black arrows indicate normal cells; white arrows indicate neuronal cells with vacuolar changes.).

The results of HE staining showed that the morphology and structure of the cerebral cortex and hippocampus of the sham operation group were basically normal. In the model group, most of the cells in the cerebral cortex and hippocampus showed pyknosis and vacuole-like changes in nuclei. In the HCC group, there were significantly more normal cells in the cerebral cortex and hippocampus than in the model group, and only a few cells showed nuclear constriction changes (Figures 7, 8).

### The Effect of HCC on Brain MVD in Rats

**FIGURE 9:**
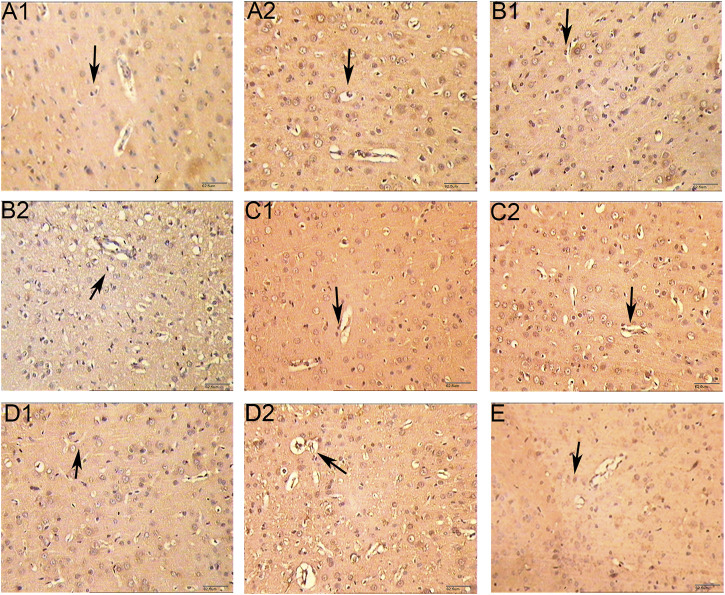
Effect of HCC on MVD at different time points in rat ischemic peripheral area (immunohistochemistry staining. 250 x A1: Model group 1 day; **(A2)**: HCC group 1 day; B1: Model group 3 days; B2: HCC group 3 days; C1: Model group 5 days; C2: HCC group 5 days; D1: Model group 7 days; D2: HCC group 7 days. E: Sham operation group. The arrow points to endothelial cells.).

The microvessels were irregular in shape and the lumen was surrounded by endothelial cells stained with brownish yellow. In the hemispheric cortex of cerebral infraction, the expression of vWF-stained vascular endothelial cells increased significantly. At 1 day after CIR, the number of microvessels in CIR group and HCC group was increased compared with the sham operation group, but the difference was not statistically significant (*p* > 0.05). Compared with the sham operation group, the number of microvessels in the HCC group increased at 3 days (*p* < 0.05); At 5 and 7 days, the number of microvessels in the HCC group increased significantly (*p* < 0.01). The CIR group also showed an upward trend. At 7 days, the MVD of the CIR group was higher than that of the sham operation group (*p* < 0.05) (Figure 9 and Table 6).

**TABLE 6:** Effect of HCC on MVD at different time points in rat ischemic peripheral area (*n* = 5, number of blood vessels/mm2, x ± s).

Group	1 day	3 days	5 days	7 days
Sham operation	-	-	-	39.0 ± 13.32
CIR model	45.0 ± 11.02	47.2 ± 11.67	57.8 ± 7.32	60.2 ± 10.37^a^
HCC	46.6 ± 12.70	60.8 ± 16.31^a^	67.8 ± 10.18^b^	67.9 ± 10.03^b^

a compared with the Sham operation group, *p* < 0.05.

b compared with the Sham operation group, *p* < 0.01.

### The Expression of Brdu and vWF

**FIGURE 10:**
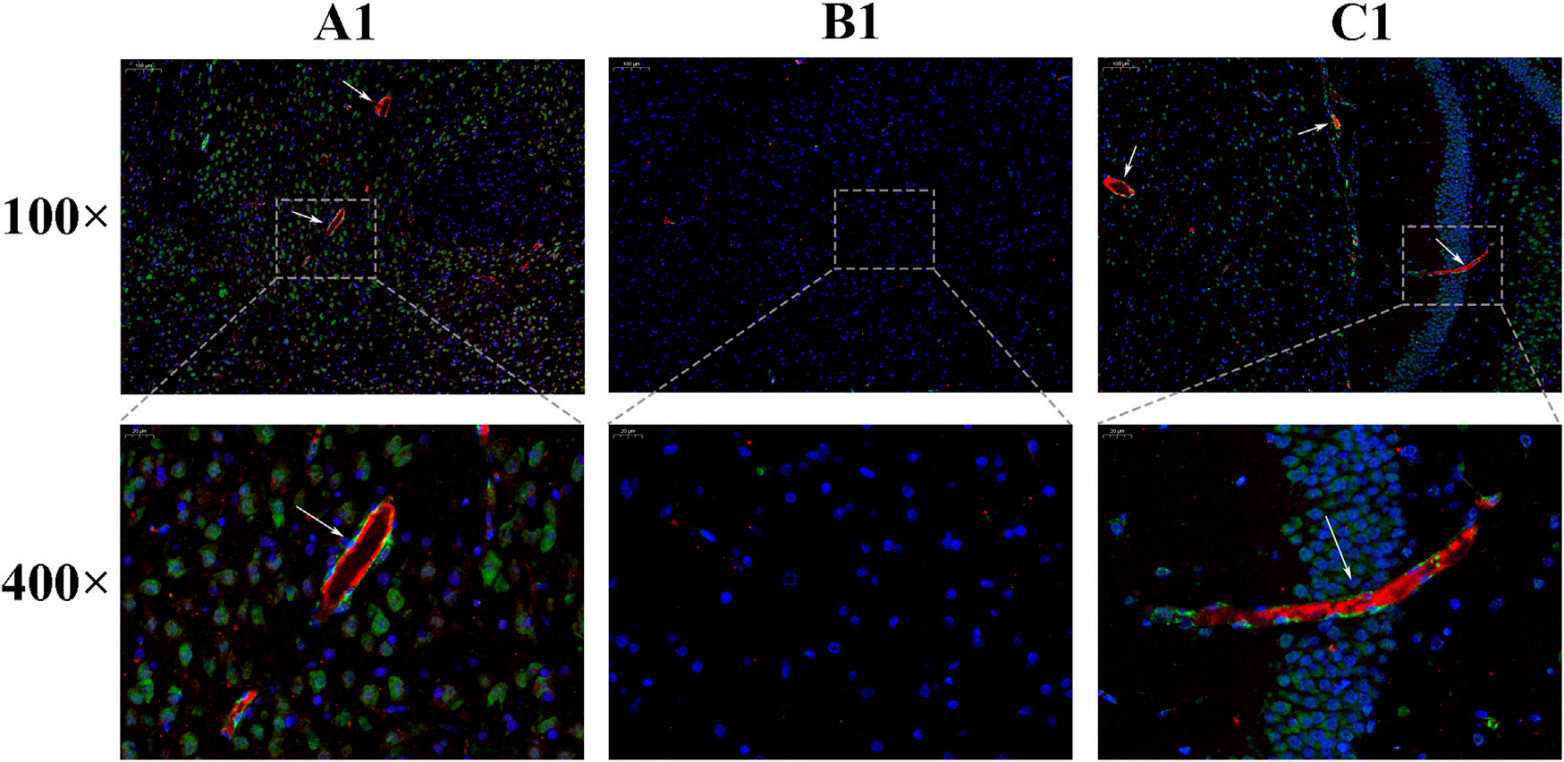
Expression of Brdu and vWF [**(A)**: sham operation group; **(B)**: Model group; **(C)**: HCC group. The areas indicated by the arrows are Brdu (green) and vWF (red)].

Under immunofluorescence, the Brdu and vWF double-stained signals appeared in the CIR group, and it was considered that there were neovascular endothelial cells. The number of positive signals in the HCC group was higher than that in the CIR group, indicating that the number of new blood vessels increased after drug treatment (Figure 10).

### The Expression of VEGF, VEGFR and HIF-1α mRNA

**FIGURE 11:**
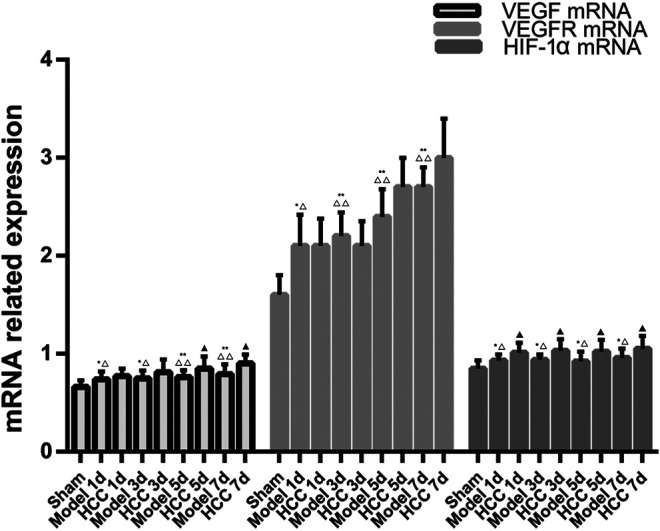
The Expression of VEGF, VEGFR and HIF-1α mRNA (compared with the normal group, *p < 0.05, **p < 0.01; compared with the Sham operation group, △*p* < 0.05, △△*p* < 0.01; compared with CIR group ▲*p* < 0.05).

Compared with the normal group and the sham operation group, the expression of VEGF, VEGFR and HIF-1α mRNA in the CIR group was enhanced (*p* < 0.01, *p* < 0.05). Compared with the model group, the expression of VEGF mRNA increased on the 5th and 7th day in HCC group (*p* < 0.05), and the expression of HIF-1α mRNA increased at each time point (*p* < 0.05). (Figure 11).

### The Expression of VEGF, VEGFR and HIF-1α Protein

**FIGURE 12:**
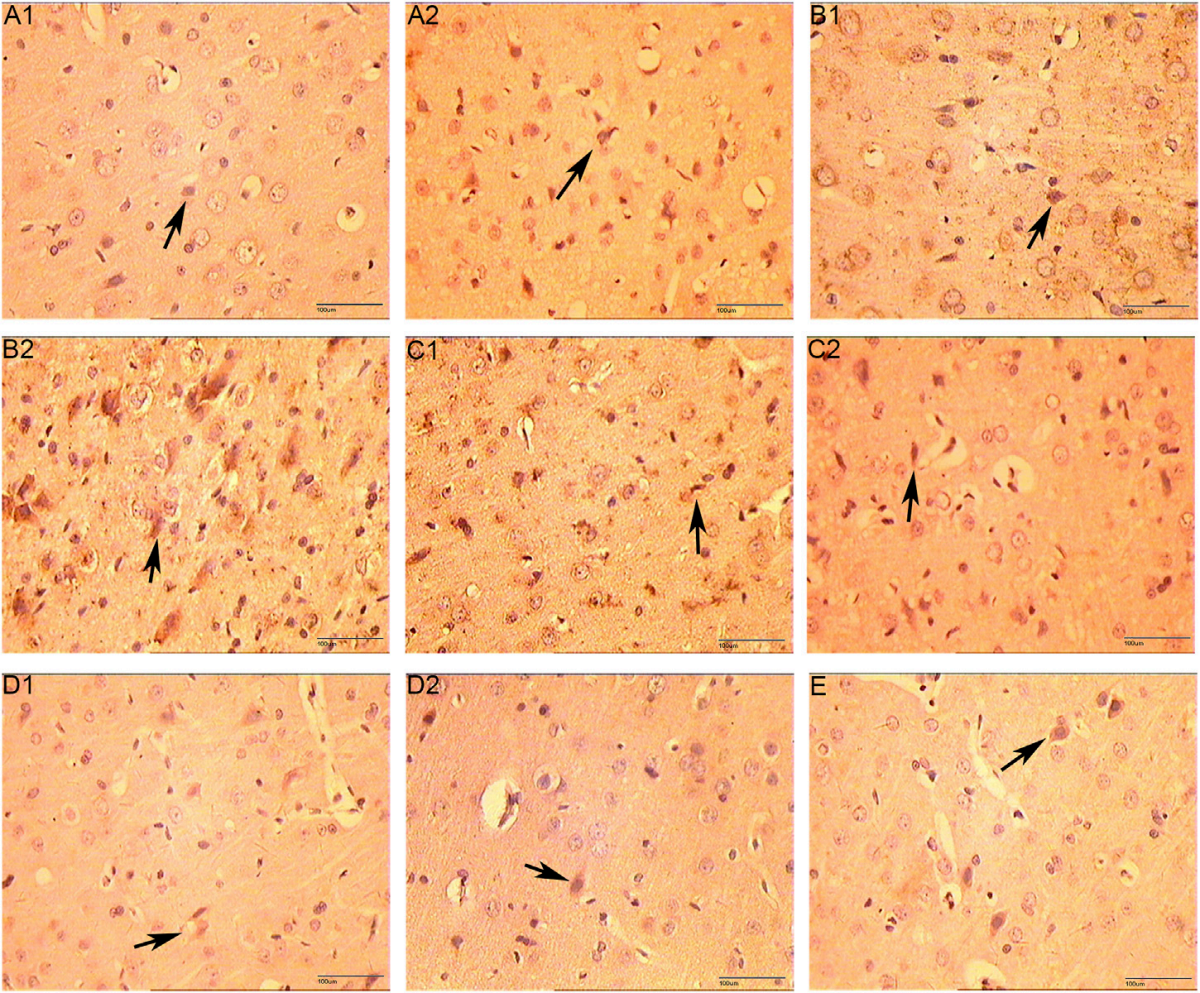
The Expression of VEGF (immunohistochemistry staining. 400 x **(A1)**: Model group 1 day; **(A2)**: HCC group 1 day; **(B1)**: Model group 3 days; **(B2)**: HCC group 3 days; **(C1)**: Model group 5 days; **(C2)**: HCC group 5 days; **(D1)**: Model group 7 days; **(D2)**: HCC group 7 days. **(E)**: Sham operation group; The arrow points to positive expression).

**FIGURE 13:**
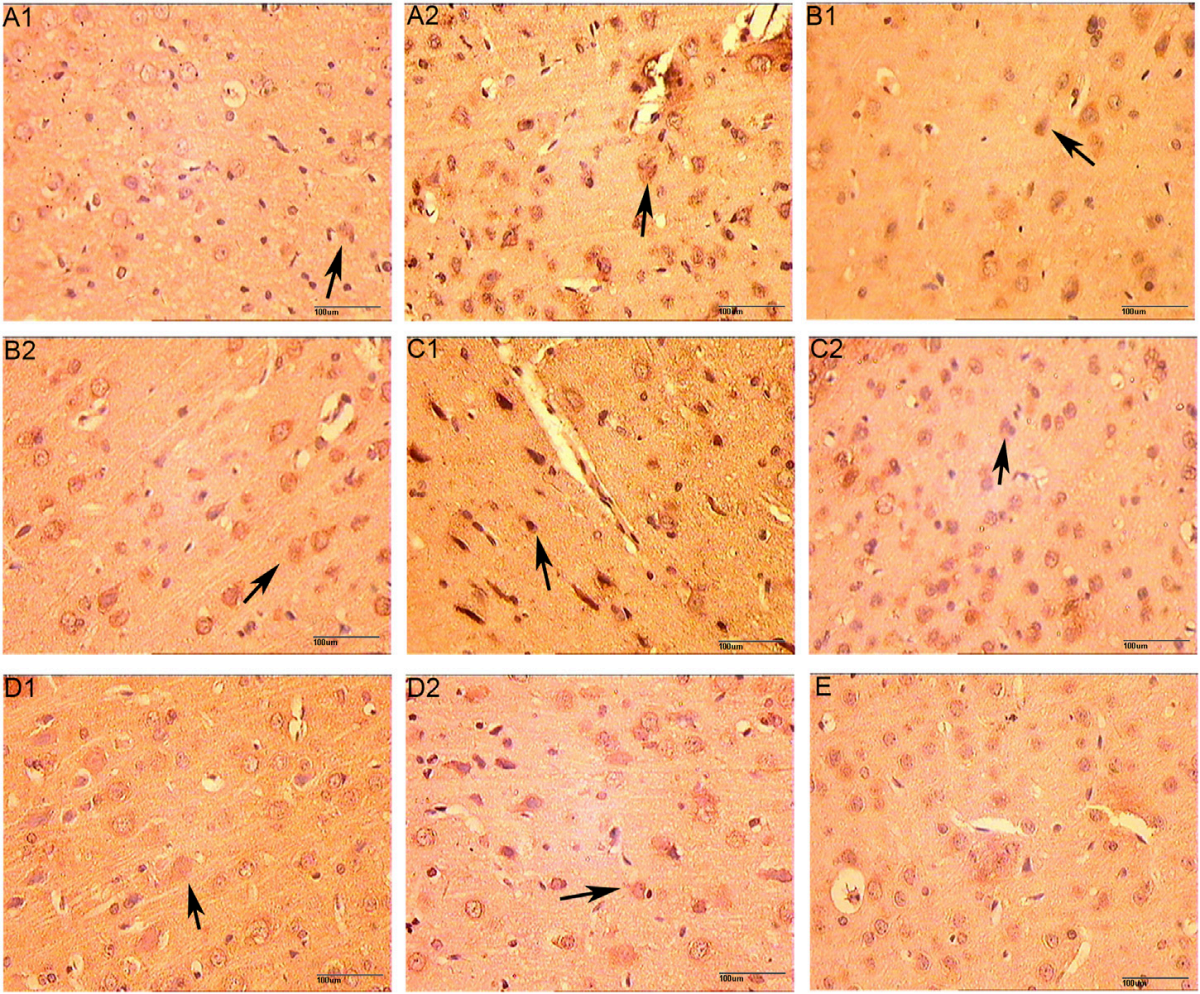
The Expression of VEGFR (immunohistochemistry staining. 400 x **(A1)**: Model group 1 day; **(A2)**: HCC group 1 day; **(B1)**: Model group 3 days; **(B2)**: HCC group 3 days; **(C1)**: Model group 5 days; **(C2)**: HCC group 5 days; **(D1)**: Model group 7 days; **(D2)**: HCC group 7 days. **(E)**: Sham operation group; The arrow points to positive expression).

**FIGURE 14:**
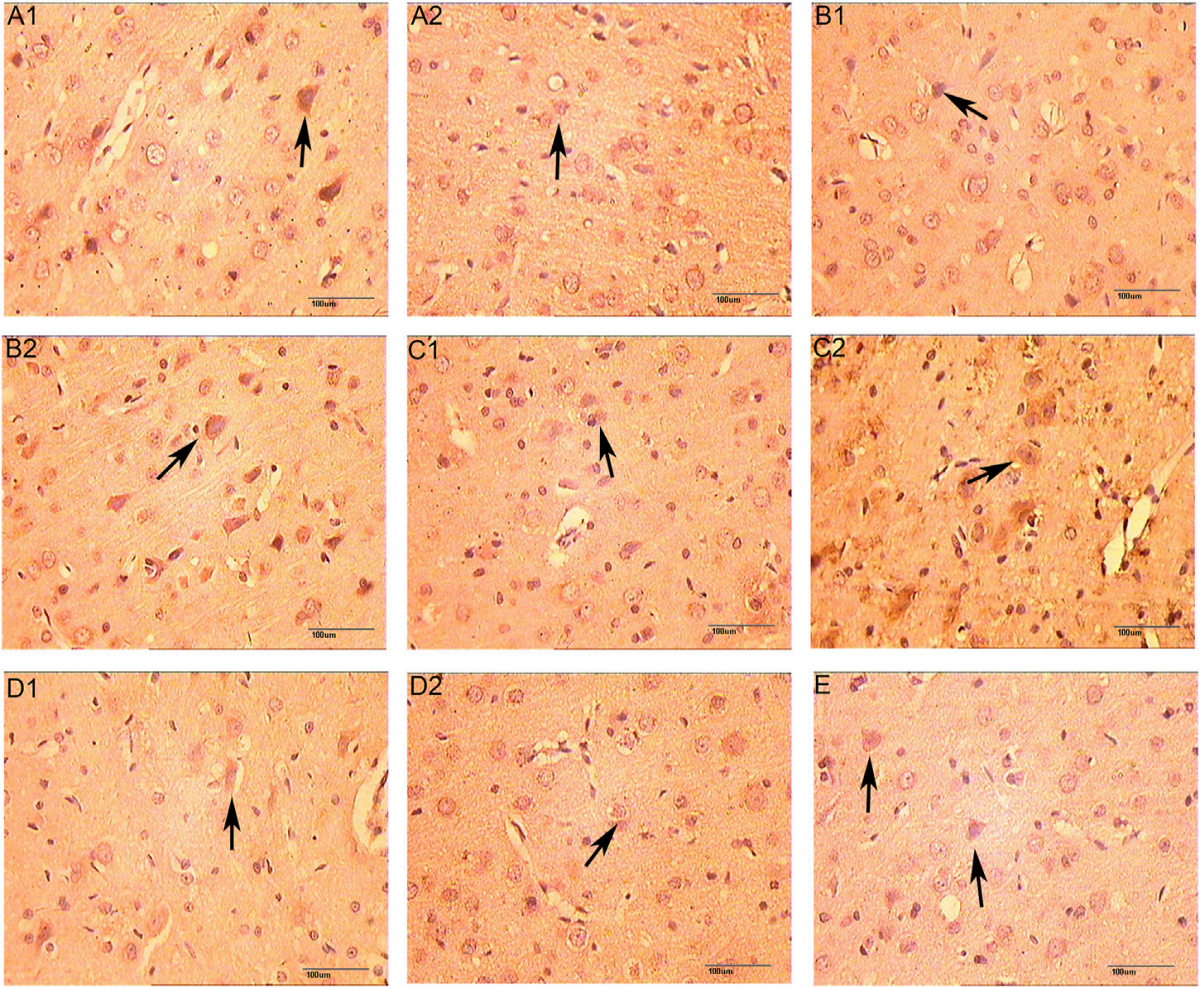
The Expression of HIF-1α (immunohistochemistry staining. 400 x **(A1)**: Model group 1 day; **(A2)**: HCC group 1 days; **(B1)**: Model group 3 days; **(B2)**: HCC group 3 days; **(C1)**: Model group 5 days; **(C2)**: HCC group 5 days; **(D1)**: Model group 7 days; **(D2)**: HCC group 7 days. **(E)**: Sham operation group; The arrow points to positive expression).

**FIGURE 15:**
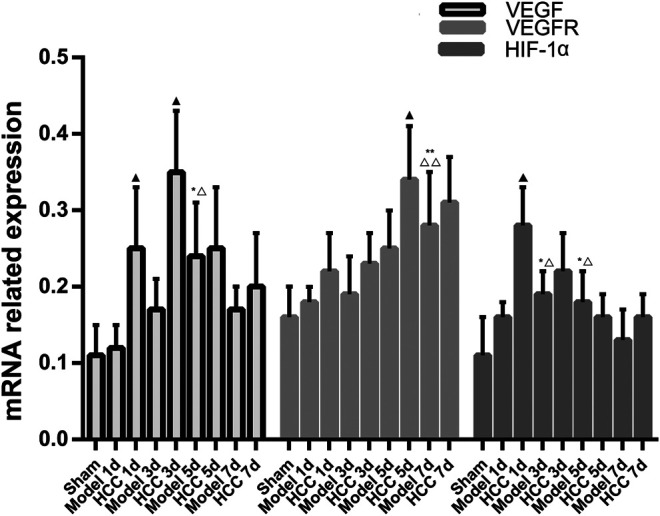
The Expression of VEGF, VEGFR and HIF-1α proteins (compared with the normal group, *p < 0.05, **p < 0.01; compared with the Sham operation group, △*p* < 0.05, △△*p* < 0.01; compared with CIR group ▲*p* < 0.05).

The positive expression of VEGF and VEGFR-2 is light yellow cytoplasm with brownish brown particles. HIF-1α is positive for brown-yellow particles mainly in the nucleus and a small amount in the cytoplasm. Compared with the normal group and the sham operation group, the expression of VEGF protein was increased on 5 days of the CIR group (*p* < 0.05), the expression of VEGFR protein was increased on 7 days (*p* < 0.05), and the expression of HIF-1α protein was increased on 3 and 5 days (*p* < 0.05). Compared with the CIR group, the expression of VEGF protein was increased on 1 and 3 days of the HCC group (*p* < 0.01), the expression of HIF-1α protein was increased on 1 day (*p* < 0.01), and the expression of VEGFR protein was increased on 5 days (*p* < 0.01). (Table 5 and Figures 12–15).

The effect of HCC on CIR angiogenesis was discovered through a systematic pharmacological method in previous section. Then, animal experiments were carried out to clarify the mechanism of HCC, and further explored the upstream pathways that HCC promotes VEGF expression. Current research found that vascular endothelial cells play an important role in vascular regeneration and maintenance of vascular morphology and function. The upstream of VEGF expression is regulated by a variety of factors. Under hypoxia, HIF-1α can promote the regeneration of blood vessels, and HIF-1α can also be used as a regulator to promote the expression of downstream VEGF (Lee et al., 2017; Chen et al., 2018; Fan et al., 2019). This experimental pharmacology section explores whether HCC can promote VEGF expression by up-regulating HIF-1α. The results showed that after CIR, the express of HIF-1α mRNA and protein increased, and HCC could further promote this effect. After CIR, the changes in VEGF and VEGFR were the same as above. Compared with the model group, the difference in HIF-1α mRNA expression in the HCC group increased on the first day after CIR, while the difference in VEGF mRNA expression appeared on the fifth day.

## Conclusion

The systematic pharmacology prediction results showed that HCC may regulate CIR-related targets (such as AKT1, MAPK1, CASP3, EGFR), biological processes (such as inflammation, platelet activation, nerve cell survival, angiogenesis, oxidative stress, neuronal axonal injury, hypoxia-induced stress, blood coagulation, calcium homeostasis) and signaling pathways (such as HIF-1, VEGF, Ras, FoxO signaling). The experiments also showed that HCC may promote angiogenesis by up-regulating the expression of HIF-1α/VEGF and VEGFR, and finally achieves the role of prevention and treatment of CIR. Hence, this research may provide new reference information for the treatment of CIR by Chinese medicine.

### Declare

The work described has not been submitted elsewhere for publication, in whole or in part, and all the authors listed have approved the manuscript that is enclosed.

## Data Availability

The original contributions presented in the study are included in the article/Supplementary Material, further inquiries can be directed to the corresponding authors.

## Abbreviations

CIR, cerebral ischemia-reperfusion injury; DL, drug-likeness; GO, Gene Ontology; HCC, Hedysarum Multijugum Maxim.-Chuanxiong Rhizoma Compound; HPLC, high performance liquid chromatography; IRI, Ischemia-reperfusion injury; MVD, microvessel density; NMDA, N-methyl-D-aspartic acid; OB, oral bioavailability; PPI, protein-protein interaction; RT-PCR, Reverse Transcription-Polymerase Chain Reaction; TCM, traditional Chinese medicine; TNF, tumor necrosis factor; TXB2, thromboxane B2.
